# Study on the influence mechanism of perceived benefits on unsafe behavioral decision-making based on ERPs and EROs

**DOI:** 10.3389/fnins.2023.1231592

**Published:** 2023-12-14

**Authors:** Shu Zhang, Qiyu Yang, Cong Wei, Xiuzhi Shi, Yan Zhang

**Affiliations:** ^1^School of Resources and Safety Engineering, Central South University, Changsha, China; ^2^School of Educational Science, Huazhong University of Science and Technology, Wuhan, China

**Keywords:** perceived benefits, unsafety behavioral decision-making, EEG, ERPs, EROs

## Abstract

**Introduction:**

Perceived benefits are considered one of the significant factors affecting an individual’s decision-making process. Our study aimed to explore the influence mechanism of perceived benefits in the decision-making process of unsafe behaviors.

**Methods:**

Our study used the “One Stimulus-Two Key Choice (S-K1/K2)” paradigm to conduct an EEG experiment. Participants (*N* = 18) made decisions in risky scenarios under high perceived benefits (HPB), low perceived benefits (LPB), and control conditions (CC). Time domain analysis and time-frequency analysis were applied to the recorded EEG data to extract ERPs (event-related potentials) and EROs (event-related oscillations), which include the P3 component, theta oscillations, alpha oscillations, and beta oscillations.

**Results:**

Under the HPB condition, the theta power in the central (*p* = 0.016*) and occipital regions (*p* = 0.006**) was significantly decreased compared to the CC. Similarly, the alpha power in the frontal (*p* = 0.022*), central (*p* = 0.037*), and occipital regions (*p* = 0.014*) was significantly reduced compared to the CC. Under the LPB condition, theta power in the frontal (*p* = 0.026*), central (*p* = 0.028*), and occipital regions (*p* = 0.010*) was significantly reduced compared to the CC. Conversely, alpha power in the frontal (*p* = 0.009**), central (*p* = 0.012*), and occipital regions (*p* = 0.040*) was significantly increased compared to the HPB condition.

**Discussion:**

The high perceived benefits may reduce individuals’ internal attention and evoke individuals’ positive emotions and motivation, leading individuals to underestimate risks. Consequently, they exhibited a greater inclination toward unsafe behaviors. However, the low perceived benefits may reduce individuals’ memory review, resulting in a simple decision-making process, and they are more inclined to make fast decisions to avoid loss. The research results can help to provide targeted intervention measures, which are beneficial to reducing workers’ unsafe behaviors.

## 1 Introduction

Unsafe human behavior is one of the main causes of accidents (Nahrgang et al., 2011; Meng, 2022). In 2021, the construction department of a bridge project recycled struts that had not been inspected for safety, and seriously violated the order of construction to save cost and raise efficiency, then caused several people to die in a large accident. In the same year, a truck driver in a certain city illegally modified his vehicle and carried people more than allowed, finally, resulting in a major accident with multiple deaths (China Production Safety Network, 2021). Unsafe behavior usually refers to deliberate choices to deviate from rules or procedures (An et al., 2023). This type of behavior is usually the result of an individual’s decision-making that weighs safety against other potential benefits (e.g., time, money, etc.) (Reason, 1990).

The perceived benefits, defined as the perception of positive outcomes resulting from a specific behavior, are considered crucial factors influencing individual behavior and decision-making (Gellman and Turner, 2013). The Theory of Planned Behavior posits that the positive or negative evaluation of a specific action is the primary determinant of their behavior (Ajzen, 1991), and perceived benefits form the foundation of this evaluation (Arora and Aggarwal, 2018). In the principles of management, high perceived benefits are often viewed as strong behavioral motivators (Carpenter et al., 2010). On the other hand, low perceived benefits can diminish the motivation to engage in a particular behavior. If an individual perceives minimal positive outcomes from a behavior, they are less inclined to engage in it. According to the Prospect Theory, individuals weigh potential risks and benefits when making decisions (Kahneman and Tversky, 1979). As perceived benefits increase, individuals are more likely to engage in these behaviors, especially when these benefits are perceived to outweigh potential risks (Aldag et al., 1980; Parsons et al., 1997). From the perspective of emotional and affective states, high perceived benefits are often associated with positive emotions and positive emotional experiences (Roeser et al., 2012; Leung, 2013). When individuals perceive significant benefits from a behavior, they might experience feelings of joy, hope, or excitement (Tamir, 2009; TenHouten, 2023). These emotional responses not only make the behavior more attractive but also play a pivotal role in driving motivation toward achieving the expected outcome (Lee and Oah, 2015). In summary, perceived benefits may shape an individual’s subjective motivations, emotional states, or other psychological factors, thereby influencing the decision-making process.

With the developments in neuroscience, many researchers have employed Event-Related Potentials (ERPs) and Event-Related Oscillations (EROs) to investigate the decision-making process. The behavior of an individual directly stems from their decision-making process (Ruggeri et al., 2019). During decision-making, individuals gather, analyze, and evaluate pertinent information, leading to a certain behavior, which is considered to be a complex cognitive process (Shadlen and Kiani, 2013; Prezenski et al., 2017). Research has found that the P3 amplitude was an important neural marker in the decision-making process, closely associated with the reallocation of attentional resources during decision evaluation (Zhang, 2009; Na et al., 2019), and was sensitive to benefits valence (Zhang et al., 2017; Wang et al., 2020). The theta and alpha oscillations in event-related oscillations, in particular, reflect cognitive and memory abilities (Klimesch, 1999; Soltani Zangbar et al., 2020). The patterns or changes in theta oscillations in the medial prefrontal regions during the decision process may be related to decision-related psychological processes and behavioral performance (Cohen and Donner, 2013). In addition, research pointed out that baseline EEG measures in particular reflected emotional traits (Davidson, 1992, 1998, 2002). Schutter et al. (2004) have confirmed that emotion could guide the decision-making process, which is reflected in changes in the alpha waves. Moreover, though beta oscillations are related to motor control (Tzagarakis et al., 2010), some studies suggest a connection between beta activity and emotional states (Hayashi et al., 2009; Charidza and Gillmeister, 2022).

Above all, perceived benefits have been proven to be a key factor influencing decision-making and behavior. However, in the context of unsafe behavioral decision-making, the profound mechanisms through which perceived benefits drive and regulate these decisions via specific psychological and neural processes remain insufficiently explored. Hence, in order to clarify the specific influence mechanism of perceived benefits on the decision-making of unsafe behaviors, this study designed a neuroscience experiment to record individuals’ electroencephalographic (EEG) signals during the whole process of decision-making in daily risky scenarios. Event-related potential techniques and time-frequency analysis methods were employed to analyze EEG temporal and spectral features. By delving into the reasons behind unsafe behavior, this study aims to provide a theoretical basis for behavioral interventions.

As mentioned above, we hypothesized that high perceived benefits and low perceived benefits will have different effects on individuals’ subjective motivations, emotional states, or other psychological factors, thereby influencing the decision-making process regarding unsafe behavior. These effects would be reflected in the P3 component, theta activity, alpha activity, and beta activity.

## 2 Materials and methods

### 2.1 Participants

Eighteen participants (11 males and 7 females), comprising undergraduate and postgraduate students, were enlisted for this compensated study. Their ages ranged from 20 to 26 years, with an average age of 23.11 and a standard deviation of 1.49. All participants were right-handed, possessed either normal vision or vision corrected with glasses/contacts, and had no history of psychosis or other mental illnesses. None had previously experienced accidents related to the study’s focus that resulted in injury or more severe consequences. And they had not consumed alcohol or caffeine 24 h before the study.

Besides, prior analysis was conducted using G*power software to pre-determine the required sample size. The statistical method used in this study was within-factors repeated measures analysis of variance, with an effect size set at 0.5, α value at 0.05, power value at 0.85, and 1 group, 3 measurements. Based on the measurement frequency, the minimum required sample size was determined to be 9.

### 2.2 Materials

**Figure 1:**
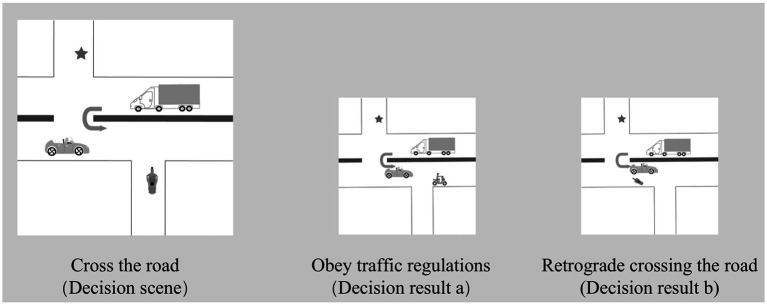
Risky scenarios and decision scenarios.

The stimuli used in the experiment were illustrations of risky scenarios, created using Adobe Illustrator. The study incorporated nine sets of these illustrations depicting everyday risk situations, including the one depicted in Figure 1. To minimize any EEG interference stemming from the image itself, a consistent grayscale design was employed, with dimensions set at 200 px × 200 px and a resolution of 72 ppi of decision scenarios.

### 2.3 Procedure

The experimental content of this study was as follows: the experiment was divided into the control, high perceived benefits (HPB), and low perceived benefits (LPB) conditions, which meant three decision conditions. The rules of the three conditions were as follows: (1) in the Control condition, when the participants made an unsafe behavioral decision and no accident occurred, there were no additional benefits or losses; (2) in the HPB condition, when the participants made an unsafe behavioral decision and no accident occurred, there would be benefits (simulation of choosing unsafe behaviors due to perceived higher benefits from such behaviors, for example, in production, regular shutdown maintenance of equipment and facilities is ignored to improve efficiency); (3) in LPB condition, when the participants made an unsafe behavioral decision, there would be losses whether or not an accident occurred (simulation of choosing safe behaviors due to perceived lower benefits from unsafe behaviors, for example, punishing illegal operation of operators in the process of operation). Before the commencement of each phase, participants were briefed on the rules and subsequently presented with a series of visual scenes. They were tasked with deciding whether to engage in unsafe behavior depicted in these scenes, with all choices being voluntary. To ensure a semblance to real-world conditions, participants could potentially instigate accidents, leading to loss. The accident probability in the test should be estimated by the participants according to experience. To facilitate experimental research, the points rules shown in Table 1 were designed to quantify each test condition by referring to relevant research (Shu, 2009). All participants in the experiment would get certain initial points. During the experiment, more points can be obtained according to the experimental rules. Of course, it was possible to lose some points. After the experiment, these points can be converted into RMB and paid to participants in the experiment according to certain rules.

**Table 1:** Points rules in the experiment.

Experiment conditions	Behavior selection	Behavior outcome	Points result
HPB condition	Unsafe behavior	No accident	10
		Accident occurs	−90
LPB condition	Unsafe behavior	No accident	−5
		Accident occurs	−105
Control condition	Unsafe behavior	No accident	0
		Accident occurs	−100

In this experiment, all the points obtained by choosing safe behaviors are 0.

**Figure 2:**
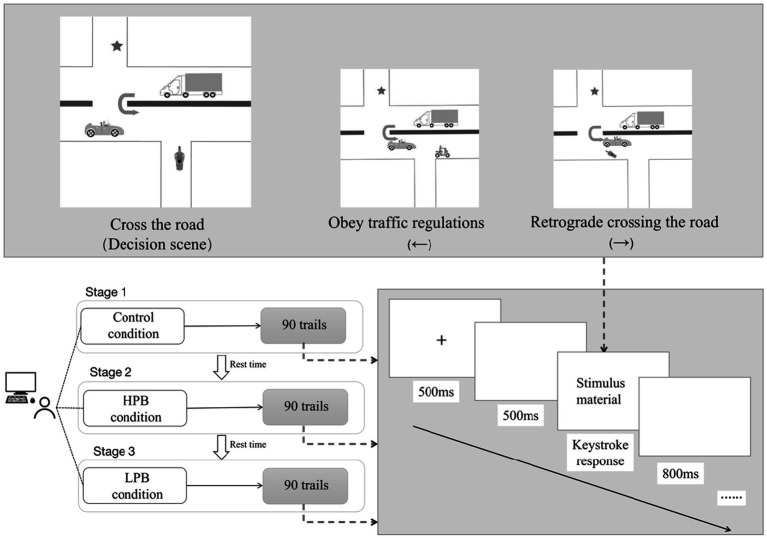
Experimental procedure.

According to the economic EEG experiment paradigm (Shu, 2009), the specific stimulus presentation process was as follows: (1) A “+” symbol appeared centrally on the screen for 500 ms, directing participants’ focus. (2) A blank screen ensued for 500 ms. (3) Risk scene stimulus materials were displayed. Upon viewing, participants pressed designated buttons to make their choices. These stimuli encompassed three images: the initial decision-making scene and two potential outcomes based on their decisions. The two outcome images, which represented safe and unsafe behavioral choices, appeared at random positions, and participants indicated their choices using left or right arrow keys. (4) Post-button press, a blank screen displayed for 800 ms, during which the program documented the selected key and the corresponding points. This was followed by a repeat of the “+” symbol. To mitigate fatigue, brief intermissions were scheduled every 30 trials (approximately 3 min). The procedural flow is depicted in Figure 2.

### 2.4 Data recording and analysis

Behavioral data were captured using the E-Prime 3.0 stimulus presentation program. The EEG data were recorded by an electrode cap (Brain Products, GER) with 32 Ag/AgCl electrodes mounted according to the extended international 10–20 system. Impedances were maintained below 5 kΩ throughout the experiment. Data were sampled at a frequency of 500 Hz. The raw EEG data were preprocessed, and components were extracted using EEGLAB 2021.0 toolbox and ERPLAB 8.2 plug-in in MATLAB R2021a environment. For raw EEG data preprocessing, the mean values of all electrodes were chosen for re-referencing, and the data were subjected to 1-30 Hz bandpass filtering as well as 50 Hz notch processing using an IIR digital filter. Artifacts resulting from cardiac activity, muscle movements, eye movements, and blinks were isolated via independent component analysis (ICA). These artifacts were subsequently removed with the assistance of the IC Label plug-in.

**Figure 3:**
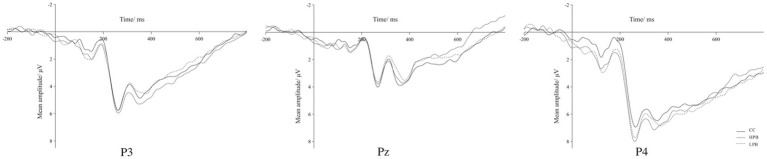
P3 Waveforms in the parietal region.

After the preprocessing, ERP waveforms for each condition were segmented based on three distinct stages. Data locked to the stimulus were divided into epochs, spanning 200 ms before and 800 ms after stimulus onset. A baseline correction was applied using the initial 200 ms of each channel. The segmented ERP waveforms were then categorized and averaged according to each condition. Subsequently, the P3 component was isolated using ERPLAB. A comprehensive analysis of the brain’s ERP waveform revealed that the P3 was primarily concentrated in the parietal region (P3, Pz, P4). As shown in Figure 3, the peak of P3 appeared around 350 ms after the stimulus was presented. In the data analysis, the peak of the P3 component was extracted within 300 ms-500 ms in the time window for statistical analysis.

**Figure 4:**
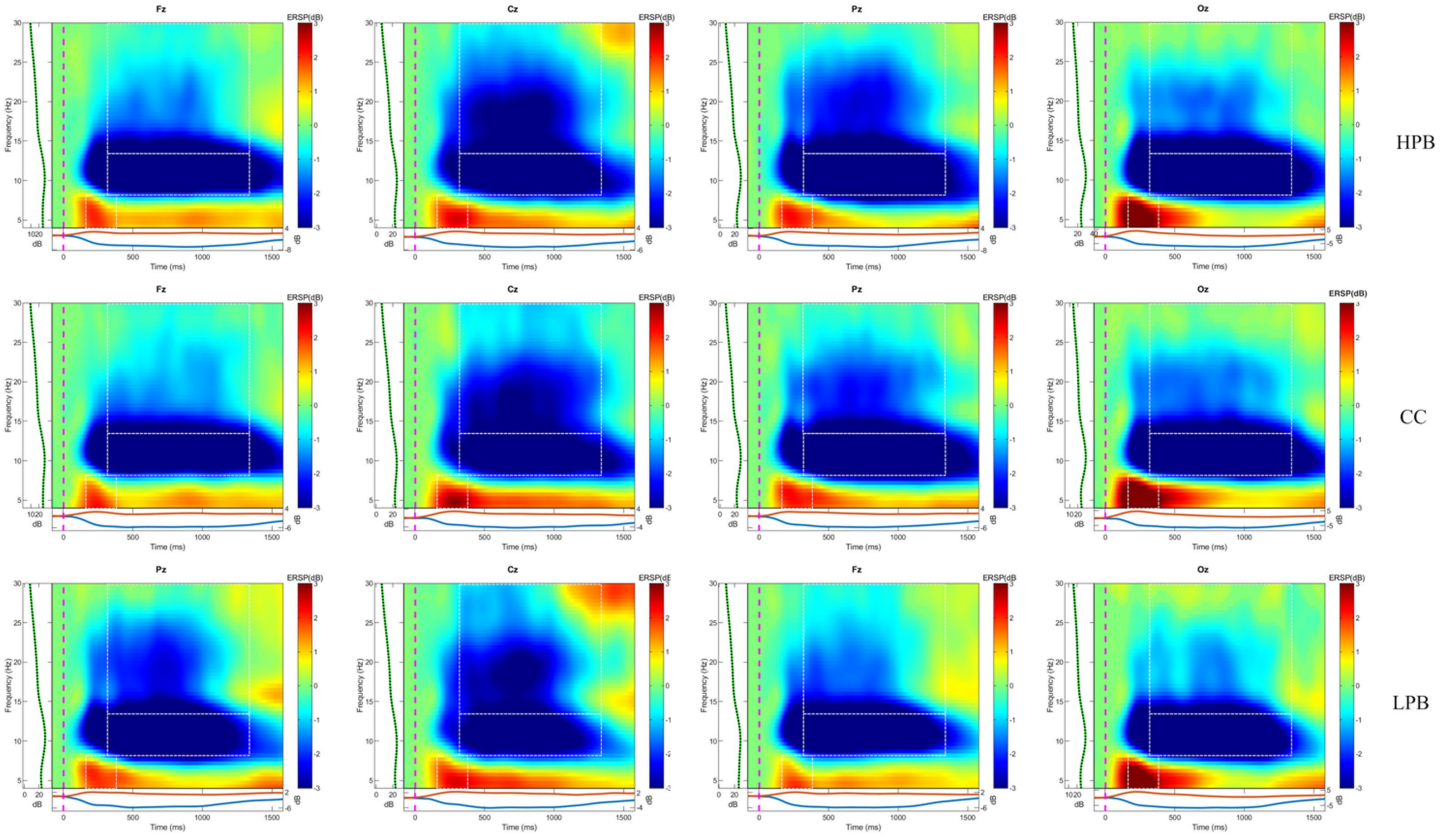
EEG time-frequency analysis results in three conditions taking Fz, Cz, Pz, and Oz electrode points as an example.

Within EEGLAB, a time-frequency analysis was conducted on the segmented stimulus-locked data, and the baseline correction was carried out using the first 500 ms of each channel. The average results of all participants (Figure 4) showed that the theta power during 200 ms-400 ms was significantly higher than the baseline, and the alpha power and beta power during 300–1,300 ms were significantly higher than the baseline. Therefore, the theta power, alpha power, and beta power were extracted in the corresponding time window from the frontal (F3, Fz, F4), central (C3, Cz, C4), parietal (P3, Pz, P4), and occipital (O1, Oz, O2) regions for statistical analysis (Zhou et al., 2023). Both behavioral data and EEG data under three experimental conditions were analyzed by One-way repeated measures ANOVA in SPSS 26. The one-way repeated measures ANOVA is a statistical method used to analyze the effect of a single independent variable across two or more different conditions on a dependent variable, where each participant is measured under all conditions. This method has been widely applied in fields such as psychology and neuroscience.

## 3 Results

### 3.1 Behavioral data

The experimental behavior data included reaction time and unsafe behavior tendencies. Reaction time referred to the time spent from the appearance of the stimulus on the screen to the participant’s key selection. Unsafe behavior tendency referred to the proportion of the number of unsafe behavior choices to the total number of trials in each experiment condition. The results of one-way repeated measures ANOVA showed that: (1) There were significant differences in the reaction time of participants across different conditions [*F*(2, 34) = 5.951, *p* = 0.013, η^2^ = 0.259, M _HPB_ = 1,217 ms, SD _HPB_ = 325 ms, M _LPB_ = 1,024 ms, SD _LPB_ = 253 ms, M _CC_ = 1,270 ms, SD _CC_ = 218 ms]; (2) There were significant differences in unsafe behavior tendency of participants across different conditions [*F*(2, 34) = 19.483, *p* = 0.000, η^2^ = 0.534, M _HPB_ = 43.64%, SD _HPB_ = 0.282, M _LPB_ = 10.93%, SD _LPB_ = 0.152, M _CC_ = 8.95%, SD _CC_ = 0.116]. The results of the *post hoc* paired comparisons showed that the reaction time of LPB condition was significantly shorter than that of CC ( *p* = 0.000*) and HPB condition ( *p* = 0.037*), but there was no significant difference between HPB condition and CC ( *p* = 0.545). The unsafe behavior tendency in the HPB condition was significantly higher than that in CC ( *p* = 0.000*) and LPB condition ( *p* = 0.000*), but there was no significant difference between the CC and LPB condition ( *p* = 0.617).

### 3.2 EEG data

The one-way repeated measures ANOVA showed that there was no significant difference in the amplitude of P3 component in the parietal region (P3, Pz, P4) across different conditions [*F*(2, 34) = 0.656, *p* = 0.525]. The beta power in the frontal region (F3, Fz, F4), central region (C3, Cz, C4), parietal region, and occipital region (O1, Oz, O2) showed no significant difference across different condition (Table 2). Both theta and alpha power showed no significant differences across each condition in the parietal region. However, the theta power and alpha power in frontal, central and occipital regions were significantly different under different conditions. After *post hoc* paired comparisons, the results shown in Table 3 were obtained. It was found that:

1. Under the HPB condition, there was a significant decrease in theta power in the central (*p* = 0.016*) and occipital regions (*p* = 0.006**) compared to the CC. Similarly, alpha power in the frontal (*p* = 0.022*), central (*p* = 0.037*), and occipital regions (*p* = 0.014*) was significantly reduced compared to the CC.
2. Under the LPB condition, theta power in the frontal (*p* = 0.026*), central (*p* = 0.028*), and occipital regions (*p* = 0.010*) was significantly reduced compared to the CC. Conversely, alpha power in the frontal (*p* = 0.009**), central (*p* = 0.012*), and occipital regions (*p* = 0.040*) was significantly increased compared to the HPB condition.
3. There were no significant differences in theta power in the frontal, parietal, and occipital regions between HPB and LPB conditions. Similarly, there were no significant differences in alpha power in the frontal, parietal, and occipital regions between the HPB and LPB conditions.

**Table 2:** Results for one-way repeated measures ANOVA of theta power, alpha power, and beta power.

	Frontal region			Central region			Parietal region			Occipital region		
	F value	*p*-value	η^2^	F value	*p*-value	η^2^	F value	*p*-value	η^2^	F value	*p*-value	η^2^
Theta power	3.678	0.049^*^	0.178	4.812	0.023^*^	0.221	2.435	0.117	0.125	7.222	0.005^**^	0.298
Alpha power	5.835	0.010^*^	0.256	5.143	0.015^*^	0.232	2.384	0.118	0.123	5.352	0.016^*^	0.239
Beta power	1.023	0.366	0.057	2.503	0.105	0.128	0.459	0.631	0.026	0.504	0.592	0.029

^*^p < 0.05, ^**^p < 0.01.

**Table 3:** Pairwise comparison outcome of theta power and alpha power in frontal, central, and occipital regions.

	Contrast condition^a^	Frontal region		Central region		Occipital region	
		Difference /dB	*p*-value	Difference/dB	*p*-value	Difference/dB	*p*-value
Theta power	1–2	0.317	0.141	0.291	0.016^*^	0.728	0.006^**^
	1–3	0.473	0.026^*^	0.372	0.028^*^	0.851	0.010^*^
	2–3	0.157	0.230	0.082	0.474	0.124	0.548
Alpha power	1–2	0.681	0.022^*^	0.517	0.037^*^	1.087	0.014^*^
	1–3	−0.012	0.952	−0.089	0.602	0.234	0.369
	2–3	−0.692	0.009^**^	−0.606	0.012^*^	−0.853	0.040^*^

^a^1, 2, and 3, respectively, correspond to the power of Control condition, HPB condition, and LPB condition; ^*^p < 0.05, ^**^p < 0.01.

## 4 Discussion

### 4.1 Behavioral data

The results showed that under the Low Perceived Benefits (LPB) condition, unsafe behaviors involved lower potential benefits, leading to a significant reduction in individuals’ decision-making time. This finding was consistent with a value-based decision-making study, which suggested that when the value of decision options was significantly higher, the ambiguity in individual choices decreased, resulting in shorter decision-making times (Senftleben and Scherbaum, 2021). When the consequences of unsafe behavior were certain losses, individuals tended to more intuitively perceive the risks associated with such behavior. Therefore, the decision-making process tends to be simpler and more direct, with individuals not needing to weigh multiple possibilities, hence leading to faster decision-making.

On the other hand, the study revealed that under the HPB condition, there was a pronounced propensity for unsafe behaviors among individuals. This trend was consistent with another behavioral study (Parsons et al., 1997). The heightened occurrence of unsafe behaviors under the HPB condition could be attributed to individuals prioritizing potential benefits over potential risks. This inclination can be explained as risk preference (Mata et al., 2018) or over-optimism bias (Sharot, 2011). In situations offering high potential benefits, individuals frequently overvalue the likelihood of positive results and undervalue or overlook the chances of adverse outcomes. As a result, high perceived benefits may lead to a misjudgment of risks, increasing individuals’ risk-seeking tendencies and resulting in more unsafe behaviors.

### 4.2 EEG data

#### 4.2.1 The influence of high perceived benefits

Under the High Perceived Benefits (HPB) condition, there was a notable decrease in frontal, central, and occipital theta power compared to the Control condition (CC). Researchers found that theta power in the midline frontal region would significantly increase in tasks involving internal attention (O’Neill et al., 2013; Ratcliffe et al., 2022). Internal attention involves the selection and regulation of internally generated information, including the cognitive control required to allocate diverse cognitive resources for balancing options in the decision-making process and deciding on the final response strategy (Miller and Cohen, 2001; Chun et al., 2011). Accordingly, our findings suggested that under the HPB condition, the individuals’ internal attention was significantly decreased compared to the CC. This implied that individuals engaged in simpler internal processing and did not utilize extra cognitive resources for decision-making thinking under the HPB condition. Moreover, another study indicated that theta waves were related to memory encoding, and successful memory encoding and recall was accompanied by a significant increase in theta activity in the occipital and central regions (Khader et al., 2010). This provided further explanation for the less thinking decision-making process observed in individuals under high perceived benefits condition, i.e., individuals called upon fewer memory resources for evaluation. We assumed the appeal of benefits likely diverted attention from potential risks, resulting in decisions predominantly driven by the perceived benefits and reduced engagement of internal thinking processes. In contrast, under the CC, individuals focused more on the potential risks and needed more cognitive resources to evaluate. This was aligned with an eye-tracking study, which suggested that the framing of the assessment in decision-making (e.g., whether it involves money or not), might affect the distribution of an individual’s attention, which in turn affected their preferences and choices (Alós-Ferrer et al., 2021).

Furthermore, a noticeable alpha power decline was observed under the HPB condition compared to the CC. Research has established that alpha waves are most prominent when the brain is in a tranquil state, and any suppression or reduction is linked with increased brain arousal (Klimesch et al., 2007; Melnik et al., 2017). This suggested that under the HPB condition, some relevant regions of the brain were activated compared to the CC. A recent review of Alpha-band oscillations and emotion pointed out that the emotional modulation of alpha ERD (enhanced desynchronization) reflected the engagement of the motivational systems when a significant stimulus is detected (Codispoti et al., 2023). Accordingly, in our research, the high perceived benefits in the decision-making process acted as a significant stimulus, triggering the individual’s motivation to pursue benefits and emotions related to benefits. Some functional MRI studies revealed that regions within the midbrain, adjacent to the dorsal tier of dopamine neurons, demonstrated increased activation when presented with stimuli signaling potential benefits (Haber and Knutson, 2010). Thus, in our experiments, high perceived benefits elicited positive emotions in individuals. Slovic et al. (2007) posited that emotional states drove human risk assessment due to the widespread use of what was known as the affect heuristic in judgment and decision-making processes. And positive emotions usually decrease the perceived probability of risk events (Zhang et al., 2023). Accordingly, we considered that the highly perceived benefits trigger positive emotions in individuals, causing them to underestimate risks and prioritize potential benefits. As a result, individuals exhibit increased unsafe behavior tendencies, which could be regarded as risk-seeking.

#### 4.2.2 The influence of low perceived benefits

Under the Low Perceived Benefits (LPB) condition, theta power in the central and occipital regions was significantly lower than in the CC. This meant that individuals performed fewer memory reviews under the LPB condition compared to the CC. We supposed that there might be more internal conflicts in individuals under the CC, such as those between experience and expectations (based on experience, a certain low-risk behavior was safe, but individuals might worry about potential adverse outcomes), leading to more internal deliberation. This was consistent with another study, which showed that individuals often experienced a significant increase in occipital theta power when performing tasks with high cognitive conflict (Fusco et al., 2022). However, under the LPB condition, given that individuals always tend to avoid identified losses (Farinha and Maia, 2021), the decision-making process became relatively simple due to the face of determined losses. Thus, because individuals focused on the information of losses, they did not engage in much empirical review. As a result, individuals made decisions on safe behaviors in a shorter time.

In this study, both beta and P3 amplitude showed no significant differences. Beta is widely believed to respond to sensory and motor components of facial and bodily actions (Ritter et al., 2009; Angelini et al., 2018). Firstly, the experimental design of this study ensured that the decision-making actions of the participants under various conditions were identical, hence the results are logical. Secondly, although in some studies beta oscillations are related to emotional cognitive processes (Cooper et al., 2013; Charidza and Gillmeister, 2022), what differentiated them from our study was that they evoked emotions through specific stimuli unrelated to the task. Thus, there is no conflict with our results. Regarding the P3 amplitude, research within the realms of economics based on economic game paradigms found that P3 amplitude is sensitive to rewards and losses (Pornpattananangkul et al., 2017; Schmidt et al., 2020), which contrasts with the findings of this study. Wu and Wang (2019) pointed out that safety benefits are not only material but also mental. Therefore, the insensitivity of the P3 amplitude to high/low perceived benefits in the experiment may be a manifestation of how safety decision-making differs from economic decision-making, which needs further research for validation.

#### 4.2.3 Neural activities model of perceived benefits affecting unsafe behavioral decision-making

**Figure 5:**
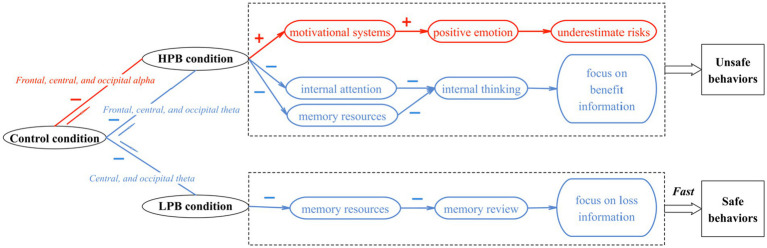
Neural activities model of perceived benefits affecting unsafe behavioral decision-making.

Following our experimental results, we found that different perceived benefits have significant effects on the decision-making process of individuals’ unsafe behaviors, which are mainly reflected in the behavioral data as well as the power changes of theta and alpha. Based on this we established a model of the influence of perceived benefits on unsafe behavioral decision-making as shown in Figure 5.

### 4.3 Implications

1. High perceived benefits can activate individuals’ motivational systems, making them more focused on pursuing benefits and subsequently underestimating the potential risks. This led to a pronounced inclination toward risk-seeking behaviors. The pursuit of high gains has always been considered a significant factor contributing to unsafe actions. In the realm of safety management strategies, it’s imperative for administrators to thoughtfully design incentive mechanisms, ensuring they do not excessively entice employees into unsafe behavior. There needs a balanced trade-off between safety and rewards. For example, a common safety management strategy is the provision of bonuses or other incentives for reaching certain milestones without any reported accidents or injuries. While this can be an effective way to encourage adherence to safety protocols, it can also inadvertently encourage the underreporting of minor incidents or the overlooking of potential risks to meet these “safe” milestones.
2. Low perceived benefits enable individuals to avoid loss quickly. This reduced the internal conflict during the decision-making process, prompting individuals to swiftly opt for decisions that avoid loss. In the context of safety management, moderate punitive measures can effectively motivate individuals to promptly avert unsafe behavior without placing undue cognitive strain. Employing such a tactic proves beneficial for timely intervention against unsafe behavior.

## 5 Conclusion

In this study, we demonstrated the significant influence of perceived benefits on individuals’ cognitive processes in decisions involving safety factors. Under the high perceived benefits (HPB) condition, individuals exhibited a significant decrease in internal attention during the decision-making process, as evidenced by a marked reduction in theta power within the frontal regions compared to the control condition (CC). Due to the attraction of high perceived benefits, the individual’s attention was focused on the benefits, resulting in decision-making that was mainly dominated by the benefits Therefore, individuals did not allocate excessive cognitive resources to contemplate potential risks. The decreased theta power in the central and occipital regions indicated fewer memory resources being engaged, which further corroborates the diminished internal attention of the individuals. Moreover, the high perceived benefits activated individuals’ brain regions associated with motivation and positive emotion, which was manifested by decreased alpha power in the frontal, central, and occipital regions compared to the CC. This led individuals to focus on pursuing benefits while underestimating potential risks. Consequently, the individual’s propensity for unsafe behavior increased significantly.

Under the low perceived benefits (LPB) condition, individuals engaged fewer memory resources, as indicated by the significantly reduced theta power in the central and occipital regions. In this scenario, due to an aversion to certain losses, individuals refrain from extensive memory recall, making fewer judgments based on experience and resulting in a more straightforward decision-making process. Therefore, the time taken to make decisions is observed to decrease.

In summary, our findings underscored the profound influence of perceived benefits on cognitive processes of decision-making and subsequent behaviors. These insights not only shed light on the neural mechanisms of unsafe behavioral decision-making but also emphasize the critical role of perceived benefits in shaping individuals’ cognitive strategies and behaviors. Such understanding is essential for designing effective interventions and strategies for unsafe behavior.

## Data availability statement

The raw data supporting the conclusions of this article will be made available by the authors, without undue reservation.

## Ethics statement

The studies involving humans were approved by Ethics Committee of School of Educational Science, HUST. The studies were conducted in accordance with the local legislation and institutional requirements. The participants provided their written informed consent to participate in this study. Written informed consent was obtained from the individual (s) for the publication of any potentially identifiable images or data included in this article.

## Author contributions

SZ, YZ, CW, and QY contributed to the conception and design of the study. XS provided research resources and platforms. CW and QY performed the statistical analysis. QY wrote the first draft of the manuscript. All authors contributed to the article and approved the submitted version.
